# Dietary inclusion of a *Saccharomyces cerevisiae* metabolite improved reproductive performance but did not affect intestinal permeability in two chicken meat breeder lines

**DOI:** 10.1016/j.psj.2024.103595

**Published:** 2024-02-27

**Authors:** Rebecca EA Forder, Nicky-Lee Willson, Joshua A Angove, Todd J McWhorter, Matthew A McQueen, David J Cadogan

**Affiliations:** ⁎ School of Animal and Veterinary Sciences, The University of Adelaide, Roseworthy Campus, Roseworthy, South Australia, 5371, Australia; † Feedworks Pty. Ltd. Romsey, Victoria, 3434, Australia

**Keywords:** broiler breeder hen, intestinal permeability, stress, inflammation

## Abstract

Gastrointestinal dysbiosis is a disturbance in mucosal homeostasis, producing low-grade chronic intestinal inflammation and impaired intestinal barrier function. It is induced by several factors, including nutrition and stress, which are both significant factors when considering current broiler breeder practices. A great grandparent (**GGP**) chicken meat line was identified displaying clinical signs characteristic of potential dysbiosis, including wet droppings and litter, in addition to reduced reproductive performance when compared to a consistently high performing line. This study aimed to determine whether the reduced reproductive performance observed in these hens was a result of dysbiosis and whether dietary supplementation with a *Saccharomyces cerevisiae* (**SC**) fermentation product would alleviate clinical signs. Dietary inclusion of SC did not influence intestinal permeability, WBC differentials, or corticosterone concentration in either the wet litter (**WL**) or high-performing (**HP**) breeder lines. Compared to hens from the HP line, WL line hens had a significant increase in intestinal permeability at 26 wk (onset of lay). WL hen heterophil counts were increased markedly at week 26 before declining. At weeks 26, 32, and 37 there were also significant increases in monocytes. Higher plasma corticosterone was also observed in WL hens at 37 wk. No significant differences in heterophil to lymphocyte (**H:L**) ratios or feather corticosterone were observed between lines. Dietary inclusion of SC supplementation to breeder diets had some benefit in regards to reducing hen mortality, improving egg production and hatchability but only in the WL line. Results from this study did not indicate that hens from the wet litter line were experiencing gut dysbiosis. Chronic intestinal inflammation may be a possible reason for the increase in intestinal permeability. These results do indicate that both breeder lines may be exhibiting physiological stress. Future investigation into the physiology and behavior around point of lay is required to find novel strategies to alleviate this stress and in turn, potentially improve welfare and production outcomes.

## INTRODUCTION

The chicken meat industry has made tremendous production gains through advanced utilization of genetic selection and nutritional understanding to ensure that chicken meat remains a low cost, desirable product for consumers (Zuidhof et al., 2015). The industry now requires new approaches to further advance efficiency and production, with greater attention now focused on broiler breeders and the mechanisms by which stressors, both environmental and nutritional, experienced by the hen can impact reproductive performance and subsequent embryo and chick development (Hynd et al., 2016). The effects of maternal stress on reproductive output is well documented in the mammalian literature, and considering current broiler breeder practices such stressors are of immense significance to industry.

Broiler breeders are subject to feed restriction to control their rapid growth rate and prevent the development of metabolic, reproductive and other obesity related problems that are often exacerbated by ad libitum feeding. While this is critical to maintaining reproductive performance, such restrictions can cause hunger and frustration leading to chronic stress in the birds. (De Jong and Guemene, 2011). Feed restriction practices in breeders have been shown to positively correlate with physiological indicators of stress such as an increased ratio of heterophils to lymphocyte (**H:L**) counts and elevated plasma corticosterone concentrations (De Jong et al., 2002; Najafi et al., 2015).

The link between stress-induced disorders and gut health is of interest in current literature. Chronic stress has been observed to impair the intestinal barrier and disrupt intestinal homeostasis. Through positive feedback mechanisms, immune, nervous and endocrine signaling pathways are continually activated, resulting in barrier disruption, increased intestinal permeability, and subsequent microbial endotoxin translocation; producing a low-grade inflammatory state (de Punder and Pruimboom, 2015). The subsequent imbalance in the maintenance of homeostasis between the intestinal mucosa and intestinal microbiota can lead to a state of dysbiosis. Interestingly, this continual low-grade chronic inflammation has been reported to affect fertility and subsequent embryo development in rodent models and human studies (Xie, et al., 2016; Fink et al., 2019; Qi, et al., 2021 ).

The growing evidence linking maternal gut health and reproduction with stress-induced disorders has highlighted the need for similar investigation in breeder hens. The major breeding companies control the pure lines of chicken meat birds, termed the “nucleus stock” (Tallentire et al., 2016). The resulting progeny produced from the nucleus stock are termed great grandparent (**GGP**) birds. Pure lines of great grandparents are then crossed to generate several lines of grandparent (**GP**) birds. The GGP lines have been selected to achieve both optimum reproductive and growth performance traits in subsequent generations, however, their ability to cope with environmental stressors, including feed restriction during peak production, has been observed to differ between lines having dramatic influence on their overall performance.

Clinical signs of dysbiosis, including increased water intake, wet, greasy droppings and dirty feathers (Teirlynck et al., 2011), have been observed in specific GGP lines as they come into lay. In poultry, noninfectious factors have been attributed to dysbiosis, including nutritional and management stressors (Teirlynck et al., 2011). The aforementioned clinical signs, in conjunction with an observed reduction in egg production and lower hatchability, has led to an in-depth investigation of these traits and application of novel strategies to alleviate their severity.

The use of dietary supplements, such as pre-, pro-, and postbiotics, to alleviate various digestive disorders including gut dysbiosis is not a new concept and is a common occurrence in clinical practice (Gareau et al., 2010; Carding et al., 2015; Wang et al., 2015). However, the influence of these supplements on maternal reproductive performance and progeny development, especially in intensive production systems, is of great interest. Yeast metabolites extracted from *Saccharomyces cerevisiae* (**SC**), are marketed as immune-health postbiotics and were originally utilized as an alternative to in-feed antimicrobials (Gao et al., 2008). The addition of SC to broiler diets reduced notable stress identifiers such as H:L ratio and plasma corticosterone concentration in response to heat stress (Price et al., 2018b) and current rearing practices (Al- Mansour, 2011). Supplementation of SC to broiler diets removed previously identified growth reductions after subjection to live-coccidiosis vaccines (Roto et al., 2017), although no noted shift in intestinal microbial populations were observed with SC supplementation (Park et al., 2017). Gastrointestinal maturation was reportedly accelerated in turkey poults subjected to yeast metabolite supplementation, where goblet cell populations, crypt depth, villus height and surface area increased following supplementation (de los Santos et al., 2007b).

There is evidence to suggest that dietary inclusion of SC has numerous benefits for bird gastrointestinal health and production. However, studies investigating the effects SC supplementation on breeder hen performance are limited. Two separate studies feeding hydrolyzed yeast (Araujo et al., 2018) and *Saccharomyces cerevisiae* (SC) extract (Kidd et al., 2013) both observed a significant increase in egg hatchability, progeny feed conversion ratio (**FCR**) as well as breast muscle yield %. These findings suggest that dietary inclusion of SC in breeder diets may provide a plausible means to introduce a cost-effective way to enhance hen health and reproductive output.

The current study aimed to investigate whether dietary supplementation prior to lay with SC would improve breeder hen health and performance. It was hypothesized that reduced reproductive output observed in a specific GGP line was a result of stress-induced gut dysbiosis. It was also hypothesized that dietary supplementation with SC metabolite would improve reproductive performance and fecal consistency through beneficial modulation of the intestinal environment, reducing physiological stress and inflammation.

## MATERIALS AND METHODS

All animal use and experimental protocols were approved by the University of Adelaide Animal Ethics Committee (S-2018-068) and the Primary Industries and Regions South Australia Animal Ethics Executive Committee (#14/18).

### Broiler Breeders

Two hundred and forty GGP broiler breeder hens from 2 different genetic lines (wet litter (WL); n = 120 and high performing (HP); n = 120), were hatched and reared at a commercial breeder facility. At 23 weeks of age, 3 weeks prior to onset of lay, birds from each line were separated into 2 treatment groups (control vs. SC). The control group was fed the facility's formulated broiler breeder diet (commercial in confidence, Laucke Mills, Daveyston, South Australia). The SC group was fed the control diet supplemented with SC (Diamond V XPC; 1000 ppm), creating 4 treatment groups (n = 60): HP-control, HP-SC, WL-control, and WL-SC. The birds were housed at the commercial breeder facility in one of their breeder sheds, with one-quarter of the shed separated into 24 smaller pens consisting of 10 hens and 1 cockerel, allowing 6 replicates per treatment. Birds were feed once daily, with total quantities (g/bird) determined the day before, based on average bird body weight and reproductive status as per commercial broiler breeder practices. Cockerels were fed separately prior to feeding the hens. Ventilation, lighting and shed temperature were all maintained to industry specifications and in accordance with the facility's commercial conditions. Mortality and egg production were recorded over the course of the trial. Hatchability of eggs was recorded from 35 to 46 wk only.

### Sample Collection

At 23, 26, 31, and 37 weeks of age 40 hens (n = 10 per treatment) were orally administered a Fluorescein isothiocyanate-dextran (**FITC-d**) solution (4.16 mg/kg Bwt). Blood samples, for both plasma and serum, were collected late-morning, prior to feeding, via venepuncture of the brachial vein two and a half hours after FITC-d administration. Sampled hens were identified with livestock marking paint to avoid resampling.

One drop of whole blood from each hen sample was placed on a glass microscope slide and a blood smear generated using the 2-slide wedge technique. The remaining blood samples were centrifuged (2000 *g* for 10 min). Plasma and serum were separated and stored at −20°C. Litter samples were collected from each pen at 23, 26, 31, and 37 wk of age. This consisted of using a bucket and obtaining litter from the front/middle/back of the pen and mixing it together before a 500 mL sub-sample was collected in a sealed container and stored at 4°C. Litter samples were analyzed for moisture content percentage (**MC%**) using a moisture content analyzer (Mettler Toledo MJ33). Pen litter samples (1.2-1.3 g) were analyzed in triplicate for all collection time points.

### FITC-d Analysis (Intestinal Permeability)

FITC-d (3–5 kDa) is a common biomarker used to assess intestinal barrier function. Serum samples were diluted 1:5 in 0.9% saline and 50 μL was pipetted onto black 96-well fluorescent plates (Corning, Sigma-Aldrich, St Louis MO). A standard curve was adapted for every plate using 8, 2-fold serial dilutions from 6,400 ng/mL to 0 ng/mL. Non-FITC-d serum was also diluted 1:5 with 0.9% saline and used as a blank (Baxter et al., 2017). FITC-d levels of diluted sera were measured at excitation wavelength of 485 nm and emission wavelength of 528 nm, gain 70 (Synergy HT, Multi-mode microplate reader, BioTek Instruments, Inc., VT). Fluorescence measurements were then compared to the standard curve, with differences in serum FITC-d (ng/mL) concentrations used as a relative index of intestinal permeability.

### H:L Counts

Whole blood smears were air-dried and fixed in methanol for 20 min following the protocol outlined by (Ballard and Cheek, 2016). Slides were later stained using a Wright-Giemsa stain in an automated slide Stainer (Siemens Hematek, Siemens, South Australia) and cover slipped. Leucocytes, including granular cells (heterophils, eosinophils, basophils) and nongranular cells (lymphocytes, monocytes), were counted at × 1000 (oil immersion lens) until a total of 100 cells per side was achieved. One slide was examined for each bird at 23, 26, 32, and 37 wk of age. H:L ratios were determined by dividing the number of heterophils by that of lymphocytes for each slide. For this study, we focused predominately on comparing the number of monocytes, lymphocytes, and heterophils.

### Plasma Corticosterone (CORT) Assay

A commercially available enzyme-linked immunosorbent assay (**ELISA**; corticosterone ELISA kit ADI-900–097, Enzo Life Sciences, Farmingdale, NY) was used to measure CORT concentration in plasma samples collected at 37 wk (peak lay), following the manufacturer's protocol for small plasma samples. Steroid displacement reagent (10 µL at 1:100) was added to 10 µL of each sample. This was vortexed and left to stand for 5 min before 380 µL of ELISA buffer was added to make a final 1:40 dilution. All samples, standards, blanks and positive and negative blanks were assayed in duplicate on a 96 well plate, and the average of each duplicate was used to calculate the final CORT concentration. Standard solutions of CORT at 32, 160, 800, 4,000, and 20,000 pg/mL were used. The absorbance of each assay was read at a wavelength of 405 nm with a plate reader (BIO-RAD, Benchmark Plus Microplate Reader, Hercules, CA)

### Feather Corticosterone (CORT) Assay

CORT was extracted from feathers using a methanol-based extraction technique previously described by Bortolotti et al. (2008). Briefly, whole feathers were weighed, and the calamus removed and re-weighed. The remaining feather was cut with scissors into ≤1 cm and pulverized using a QIAGEN Tissuelyser (Hilden, Germany), at a frequency of 30/s in 3 separate 30-s intervals to avoid overheating.

Feather samples were weighed (30 mg) and placed into low binding 15 mL glass borosilicate tubes. Ten mL of HPLC-grade methanol was added to each sample which was then shaken in a tube shaker (Ratek, Orbital Tube Shaker, Ratek Instruments Pty Ltd, Victoria, Australia) for 30 min at room temperature, followed by overnight incubation in an orbital shaker (Ratek, Orbital Shaker Incubator, Ratek Instruments Pty Ltd, Victoria, Australia) at 50°C and 300 rpm. Feathers were separated from the methanol using a vacuum filtration system (GracePure 12-Port Vacuum Manifold, Columbia, MD), using a frit disk (a porous disk made of plastic, to filter out solid particulate matter while allowing liquids to pass) and Whatman #1 filter paper placed on top of the disk. The feather remnants, original extraction tube, and filtration tube were washed twice with 1 mL of additional methanol, which was added to the extractant. The methanol extracts were placed in a 50°C water bath and evaporated under a nitrogen stream. Once evaporated extract residues were resuspended in 500 µL of PBS (pH 7.6) and split into two 250 µL aliquots. Reconstituted samples were frozen at –20°C until analyzed. The amount of CORT in samples was measured using the same ELISA kit and standard protocol as the plasma samples, however, these samples were run through the ELISA assay undiluted with assay buffer.

### Statistical Analyses

Analysis of experimental data was performed by linear mixed model analysis for independent factors, line and diet, following the procedures of IBM, SPSS Statistics 25 program (Armonk, NY). The data were checked for normality by the Shapiro–Wilk test. Non-normalized data were analyzed using nonparametric tests including Mann-Whitney U and Kruskal-Wallis. White blood cell count data were analyzed by mixed model generalized linear model analyses for independent factors, line and diet, with either linear or negative binominal regression target distribution. H:L ratios were logged for normality and analyzed by a mixed model linear analysis. Additionally, mortality and hatchability data were analyzed using a Pearson's chi-square test with the factors of line and diet fitted. For egg production data, a General Linear Model (**GLM**) procedure for repeated measures were used. A probability level of less than 5% (α = 0.05) was deemed as statistically significant.

## RESULTS

### Mortality (%)

The addition of SC reduced total mortality. However, the difference was only statistically significant within the WL line (control = 18.3% mortality; SC= 5%, *P* = 0.04) vs. HP line (control = 11.9% mortality; SC = 6.7%; *P* = 0.328*)*. No differences in overall mortality between lines were observed (HP = 10.8%; WL = 9.2%; *P* = 0.683).

### Egg Production

**Figure 1:**
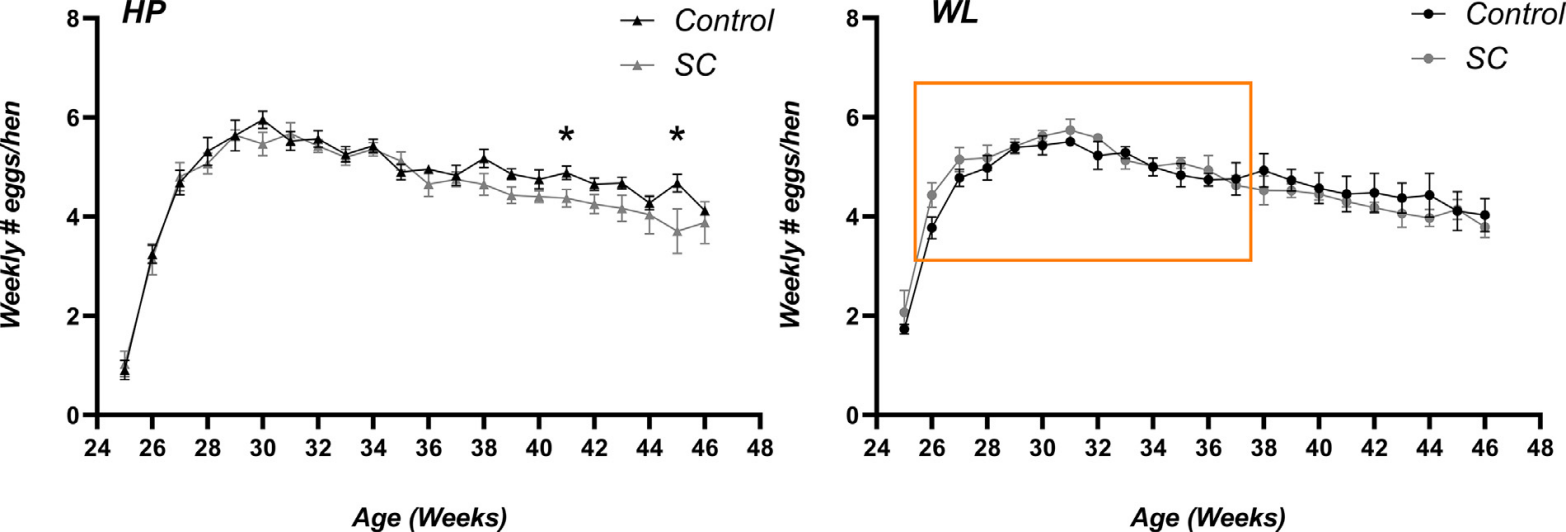
Egg production in hens from the high-performing line (HP) and wet litter line (WL) fed with and without SC. Values are means ± SEM.

Feeding HP hens SC did not significantly increase egg production at any time point. At week 37, egg production started to decline in both lines, with HP-SC hens declining slightly more in production (Figure 1). Interestingly, in WL hens, although not statistically significant, inclusion of SC tended to increase egg production between 26 and 37 wk of age (orange box, Figure 1). This contributed to a 4.7% increase in egg production in total over this period (mean eggs/bird, control = 4.98 ± 0.14 vs. SC = 5.16 ± 0.11).

### Hatchability (%)

**Figure 2:**
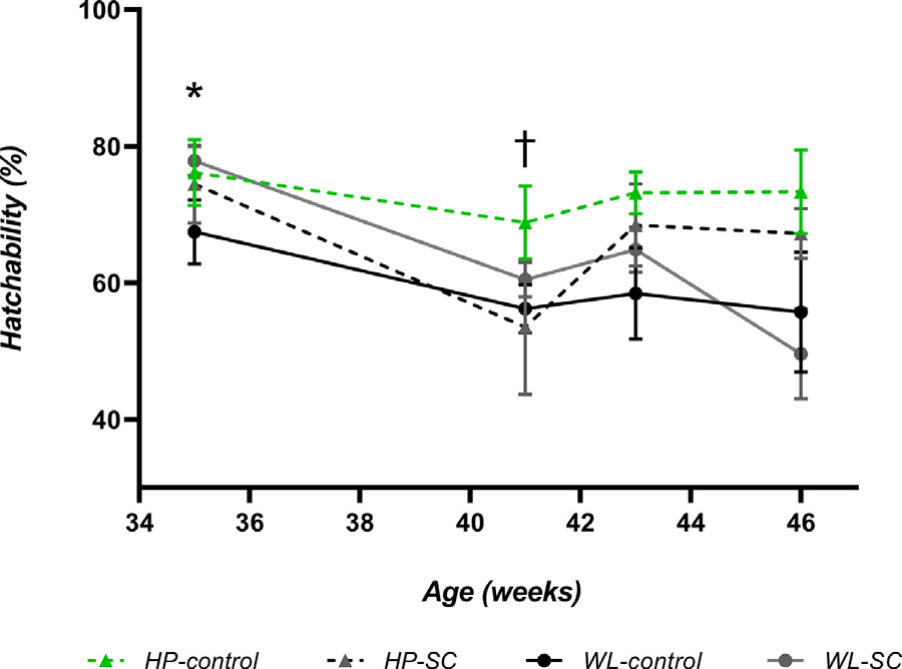
Percentage hatchability of eggs from both HP and WL hens fed with and without SC. Values are means ± SEM. Week 35 =1045 eggs set; week 41 = 538 eggs set; week 42 = 1387 eggs set, and week 46 = 862 eggs set. *denotes significance (*P* < 0.05) within WL only. ^†^denotes significance (*P* < 0.05) within HL only.

Eggs from HP hens had a greater hatchability between 35 and 46 wk compared to eggs from WL hens (70.75% vs. 62.41%; *P* < 0.0001) Hatchability in WL-SC hens was increased from week 35 to 43, but this was only statistically significant when eggs were hatched from 35-wk-old hens (*P* = 0.004; Figure 2). Dietary inclusion of SC did not positively affect hatchability in HP hens at any time point, at 41 wk, hatchability was significantly lower in hens receiving SC (*P* = 0.005*;* Figure 2)*.*

### Litter Moisture

**Figure 3:**
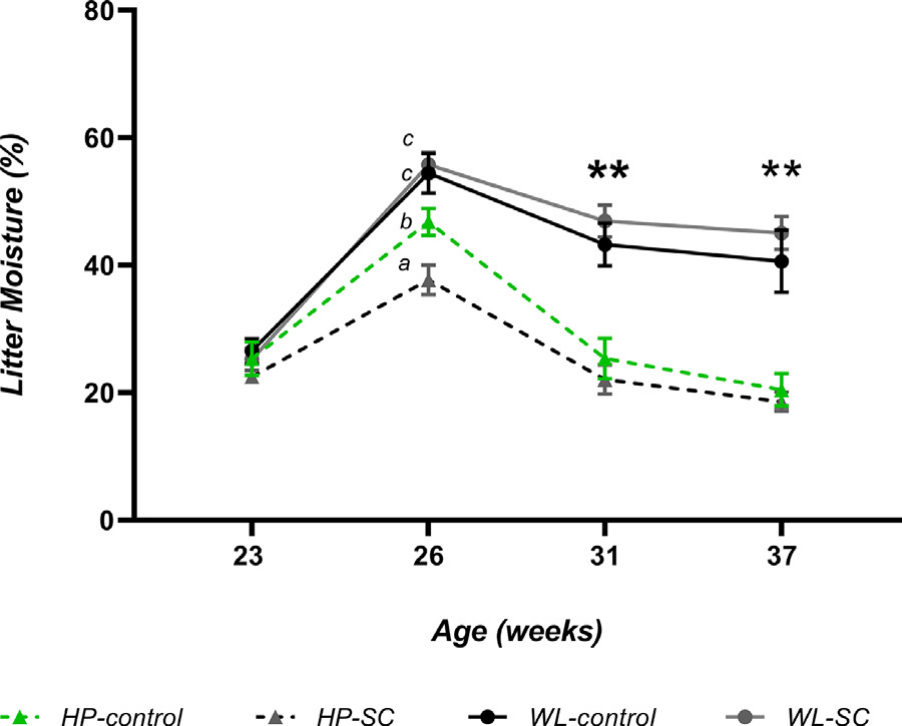
Litter moisture content (%) from pens containing both breeder lines (HP and WL) fed with and without SC. Values are means ± SEM. Week 26, different letters denote a significant difference *P* < 0.05. **denotes significance (*P* < 0.01) between lines only.

Litter moisture increased in both lines from week 23 to week 26 (onset of lay). WL hens consistently had significantly higher litter moisture from 26 wk onwards compared to the HP line. In HP hens, litter moisture continued to reduce after 26 wk. The addition of SC reduced litter moisture in HP-SC hens at week 26 only (*line x diet interaction, P* = 0.043), this was not observed in either WL-C or WL-SC hens at any time point (Figure 3).

### Intestinal Permeability (FITC-d)

**Figure 4:**
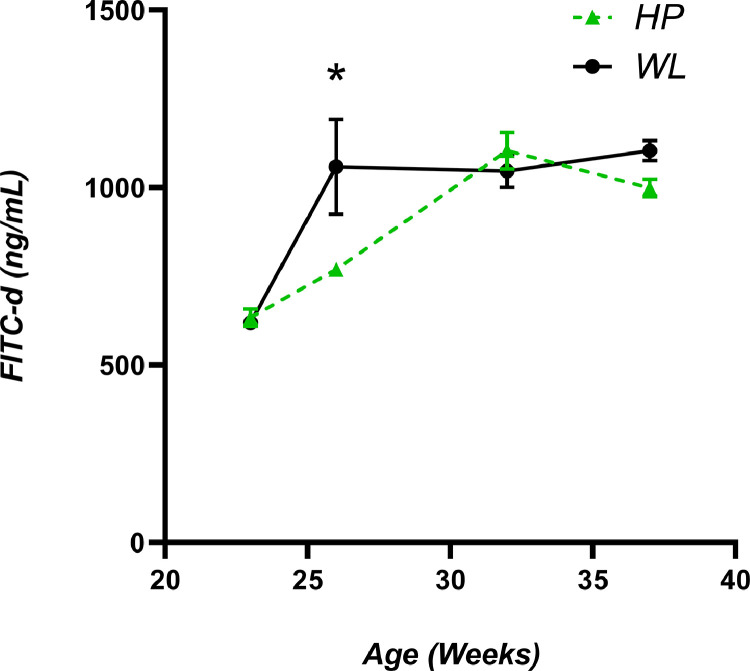
Detection of serum FITC-d (ng/mL) as a marker of intestinal permeability of HP and WL lines, at 23, 26, 32, and 37 wks of age. Values are means ± SEM. *denotes significance (*P* < 0.05).

Dietary inclusion of SC fed from 23 wk did not affect intestinal permeability in either breeder line at 26, 32, or 37 wk (*P* = 0.209; *P* = 0.428 and *P* = 0.240 respectively), both control and SC data was then combined and analyzed to detect any differences between breeder lines. FITC-d results from 26-week-old birds displayed a significant sharp increase in intestinal permeability in WL compared to HP hens (1058.89 ± 134.04 vs. 769.21 ± 20.83 ng/mL), which then plateaued. (Figure 4; *P* = 0.04). HP hens also displayed an increase in intestinal permeability at week 32 (peak lay) and week 37, which were similar to values obtained from WL hens.

### WBC Differentials

No significant effects were identified with the inclusion of SC (all *diet x line interactions* and *diet P* > 0.05) for the WBC differential counts, with the following data combined and presented to examine the effects between HP and WL lines.

### Heterophil Counts

**Figure 5:**
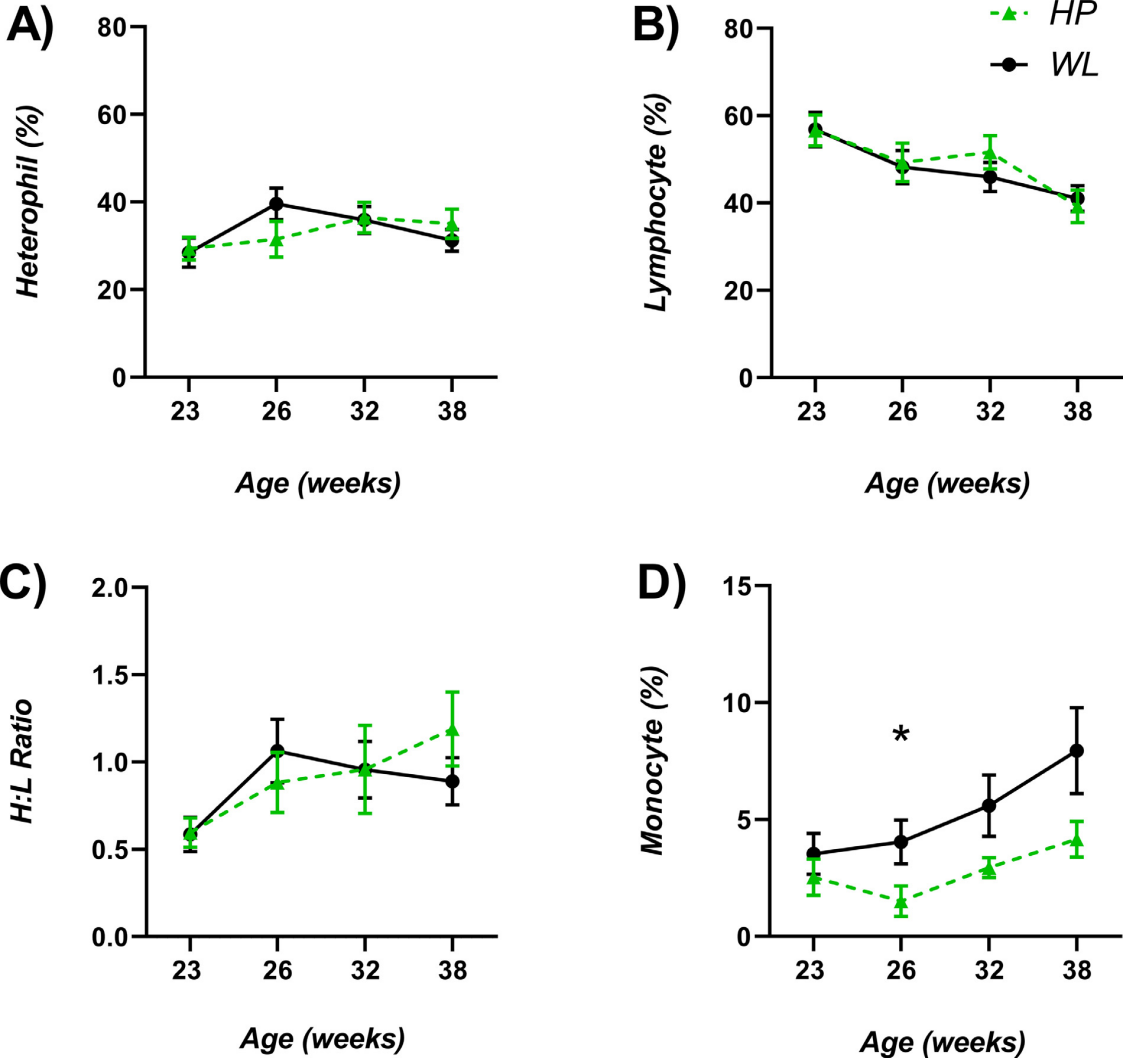
Heterophil % (A), Lymphocyte % (B), H:L ratio (C), and Monocyte % (D) in whole blood as markers of stress and inflammation in breeder hens from 2 genetic lines; HP (n = 20) and WL (n = 20), at 23, 26, 32 and 37 wk of age. Values are means ± SEM. *denotes significance (*P* < 0.05).

Both HP and WL lines had similar heterophil counts beginning at week 23 (Figure 5A; *P* = 0.827). The heterophil levels of WL hens (expressed as % of 100 WBC total count) increased markedly from 28.40 ± 3.36 at week 23 (prelay) to 39.55 ± 3.63 at week 26 (onset of lay), compared to HP hens (31.50 ± 4.10), although highly variable and not significant (*P* = 0.152). WL heterophil count then decreased at week 32 and week 37. HP heterophil levels continued to increase gradually to a maximum at week 32 (36.44 ± 3.47) before plateauing by week 37 (*P* = 0.404).

### Lymphocyte Counts

Lymphocyte counts were indistinguishable at week 23 between HP (56.65 ± 3.52) and WL (56.80 ± 4.00; *P* = 0.997; Figure 5B). The greatest difference between the 2 lines was observed at week 32. The counts in HP hens had increased from the previous week 26 timepoint to 51.63 ± 3.84, compared to WL hens, 45.94 ± 3.38, however the differences were not significant (*P* = 0.307). By week 37, overall lymphocyte counts had reduced with no difference between lines observed (*P* = 0.712).

### H:L Ratio

The H:L ratios for both HP and WL lines were highly variable with no significance detected at any timepoint (Figure 5C). Both lines increased from week 23 to 26. From there onwards the H:L ratios for WL remained constant, whereas HP reduced at week 32 (*P* = 0.376*;* Figure 5C), before increasing again at week 37 (*P* = 0.557). In addition, there was no relationship between week 37 plasma CORT concentration and week 37 H:L ratio (*r* = 0.05*; P* = 0.076).

### Monocyte Counts

At week 23, monocytes (%) in WL hens (3.53 ± 0.87) and HP hens (2.53 ± 0.78) were similar (*P* = 0.414). HP hen monocyte numbers gradually increased at each time point, as did WL hens, but to lesser extent. Notably, at week 26 (*P* = 0.015), there was a significantly higher percentage of monocytes in WL compared to HP hens (Figure 5D). Although remaining higher for the remaining timepoints, no significance was detected at week 32 (*P* = 0.123) or week 37 (*P* = 0.093), with high variation within the count data.

### Plasma Corticosterone (ng/mL)

**Figure 6:**
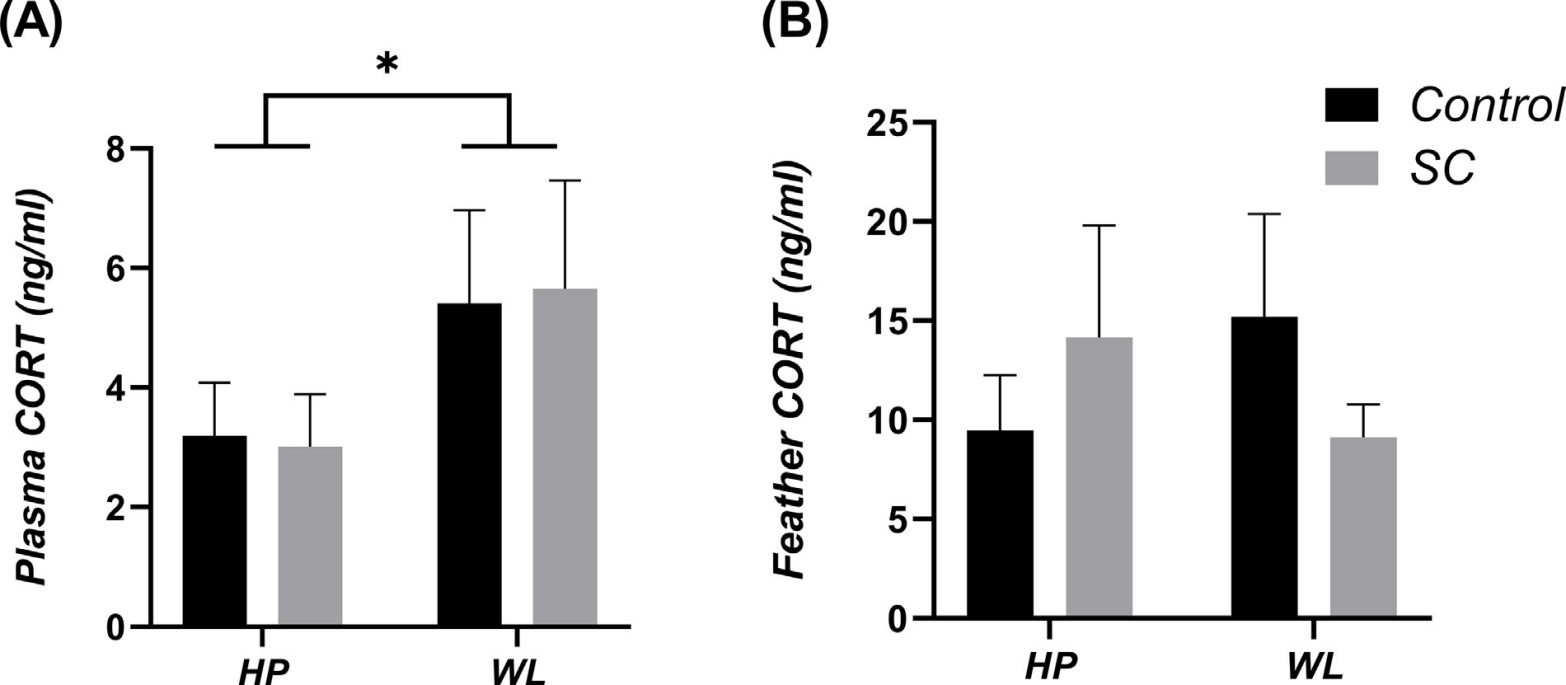
(A) Plasma corticosterone (CORT) concentrations of 37-week-old breeder hens from HP and WL lines, +/- dietary inclusion of SC. *denotes significance (*P* < 0.05). (B) Feather corticosterone (CORT) concentrations of 37-week-old breeder hens from HP and WL lines, +/- dietary inclusion of SC. Values are means ± SEM.

Large variation in plasma CORT at 37 wk was observed within WL-control and WL-SC hens. WL hens had significantly higher plasma corticosterone compared to HP hens (5.05 ± 0.85 vs. 2.98 ± 0.47 ng/ml; *P* = 0.04). The addition of dietary SC had no effect on plasma CORT concentration (Figure 6A). No interaction between line and diet was observed.

### Feather Corticosterone (ng/mL)

Feather CORT at week 37 had a large amount of variation between breeder hen line and dietary treatment groups (0.3 ng/mL to < 20 ng/mL; Figure 6B), especially in the WL-Control hens. There were no significant differences in feather CORT concentration between lines. The addition of SC tended to lower feather CORT in comparison to control in both lines (SC, 7.91 ± 1.29 vs. Control, 9.82 ± 1.30 ng/mL) but was not statistically significant (*P* = 0.558; Figure 6B). There was no significant interaction observed between breeder line and dietary inclusion of SC.

## DISCUSSION

The current study hypothesized that SC supplementation would improve reproductive performance and fecal consistency of an underperforming GGP line, through beneficial modulation of the intestinal environment, reducing physiological stress and inflammation. SC fed to the WL line may be beneficial in regards to egg production and hatchability during early-mid lay, but requires further investigation given the design of the experiment, number of birds and only 1 cockerel/pen. Improved hatch percentage was similar to that reported by Kidd et al. (2013), who observed an increase in hatchability from parent hens at 32 and 39 wk of age when hens were fed SC.

The addition of dietary SC (1,000 ppm) to breeder hen diets at 23 wk of age did not affect gut permeability and did not improve the wet litter issue produced by WL birds. Dietary inclusion of SC has shown to promote digestive health, improve immune function and gut morphology (de los Santos, et al., 2007a), but the specific mechanisms of action remain to be explored. The effects of addition of SC to breeder diets in this instance may purely be nutritional: given that breeder hens are severely feed restricted, an additional 15% protein source at 1,000 ppm may explain the improvement in egg production and hatchability during early-mid lay, and thus may not act by modulating the gastrointestinal environment. In order to determine if SC is indeed having a modulating effect on the gastrointestinal function, feeding birds SC from hatch and tracking gastrointestinal structure and microbiota throughout the life of the flock may be beneficial. There is early evidence to suggest at-hatch administration of probiotics can significantly alter gut microbial community structure and concurrent immune development compared to later dietary interventions, including at onset of lay (Baldwin, et al., 2018; Willson, et al., 2019).

Intestinal permeability increased in both lines as they came into lay, however the HP line did not display the dramatic increase in litter moisture produced by the WL line. There is evidence to suggest that permeability does increase with acute and chronic stress (Söderholm and Perdue, 2001) which may explain the increase in intestinal permeability during peak-lay in HP hens, although studies in birds are limited. The FITC-d levels appear to decrease from week 32, which if continued, could correlate with resolution of an acute response that caused a rapid but relatively short-lived increase in intestinal permeability. The sudden increase in intestinal permeability at 26 wk as WL hens were coming into lay may have long term and permanent effects on this line. In human studies, there is evidence to suggest that leaky gut allows passage of immune triggers and illicit innate immune response, (Tersigni et al., 2018), if such a response exists in poultry, this could explain the higher fecal water output in the WL line and potentially lower overall egg production.

From the results of the current study, it could not be determined whether inflammation alone is responsible for WL's wet excreta and reduced performance. There are indications however, particularly from the heterophil counts, that acute inflammation may be present in WL hens most severely at week 26 (onset of lay), with increased monocyte, and lesser extent reduced lymphocyte data suggesting a subsequent shift to chronic inflammation, which has been observed in birds (Shini, et al., 2010; Nazar and Marin, 2011) and mammals, including humans (Murray, et al., 2001; Maydych et al., 2017). Thus, quantification of a larger profile of immune biomarkers including T and B lymphocyte differentiation is recommended.

H:L ratios and plasma corticosterone were highly variable between birds and treatments, therefore no definitive links with intestinal permeability and production parameters could be made. H:L ratios for both lines were higher when compared to previous broiler studies (Price et al., 2018a; Weimer et al., 2018) so our breeder hens may be experiencing stress, especially during lay. It has been reported that corticosterone deposition in feathers parallels modulation of the hypothalamo-pituitary-adrenal axis (endocrine regulation of the stress response) (Bortolotti, et al., 2008). Birds may down‐regulate the adrenocortical response to stress during the lay period as a strategy to maximize reproductive success (Wingfield and Sapolsky, 2003; Bortolotti, et al., 2008). The increase in plasma corticosterone, and to a lesser extent feather corticosterone in the poor performing, wet litter line, may be attributing to their poorer reproductive performance, as they may be unable to effectively modulate their stress response.

The use of the term “stress” remains difficult to define as it may either indicate environmental constraints that decrease the performance of individuals or flock (stressor), or it may refer to an individual responding to the stressor (stress response). We cannot definitively state that WL line hens are more “stressed” than HP line hens, however our results do suggest that as a flock, the huge variation observed for both plasma corticosterone, H:L ratios and intestinal permeability may account for the poorer performance of the flock as a whole. A larger scale trial, sampling a greater number of birds, would be required to determine if this is indeed the case.

No correlation between intestinal permeability, H:L counts and inflammatory markers could be obtained, thus results from this study cannot definitively conclude that hens from the poor performing line are experiencing gut dysbiosis. In future, it would be beneficial to necropsy culled hens for histopathology to examine potential localized gastrointestinal inflammation, to definitively diagnose dysbiosis.

Concurrently, in-depth microbial analyses are also required to determine if the transition from pre to early-lay causes permanent alteration in intestinal microbiota and whether such alterations are responsible for increase in intestinal permeability and wet litter. The gut microbiota is hypothesized to be modulated by the stress response, which, via the brain-gut-microbiota axis, feeds back and influences the inflammatory response through microbial metabolite regulation of cytokine production and influence on enteric gastrointestinal barrier function (Rubio and Huang, 1992; Powell et al., 2017). However, the links between gut dysbiosis and low-grade chronic inflammation in broiler breeder hens remain largely unexplored.

The mechanisms by which wet excreta, and consequently wet litter, is occurring in these WL hens and whether it is indeed linked to gut dysbiosis warrants further study. The observed traits could not be alleviated with the dietary supplementation of SC from 23 wk of age. Future, in-depth, investigation of such on-farm issues will ultimately optimize breeder health and performance at all breeder tiers, and thus improve overall farm and industry productivity.
